# Supplementary material for: Generative AI extracts ecological meaning from the complex three dimensional shapes of bird bills
Source: PLoS Comput Biol. 2025 Mar 17;21(3):e1012887. doi: 10.1371/journal.pcbi.1012887 (PMC11940575; doi:10.1371/journal.pcbi.1012887)
Supplement: S1 Fig — The stage 2 VAE histograms and QQ-plots show distinctively increased conformation to Gaussian distributions. (PDF) [file pcbi.1012887.s001.pdf]

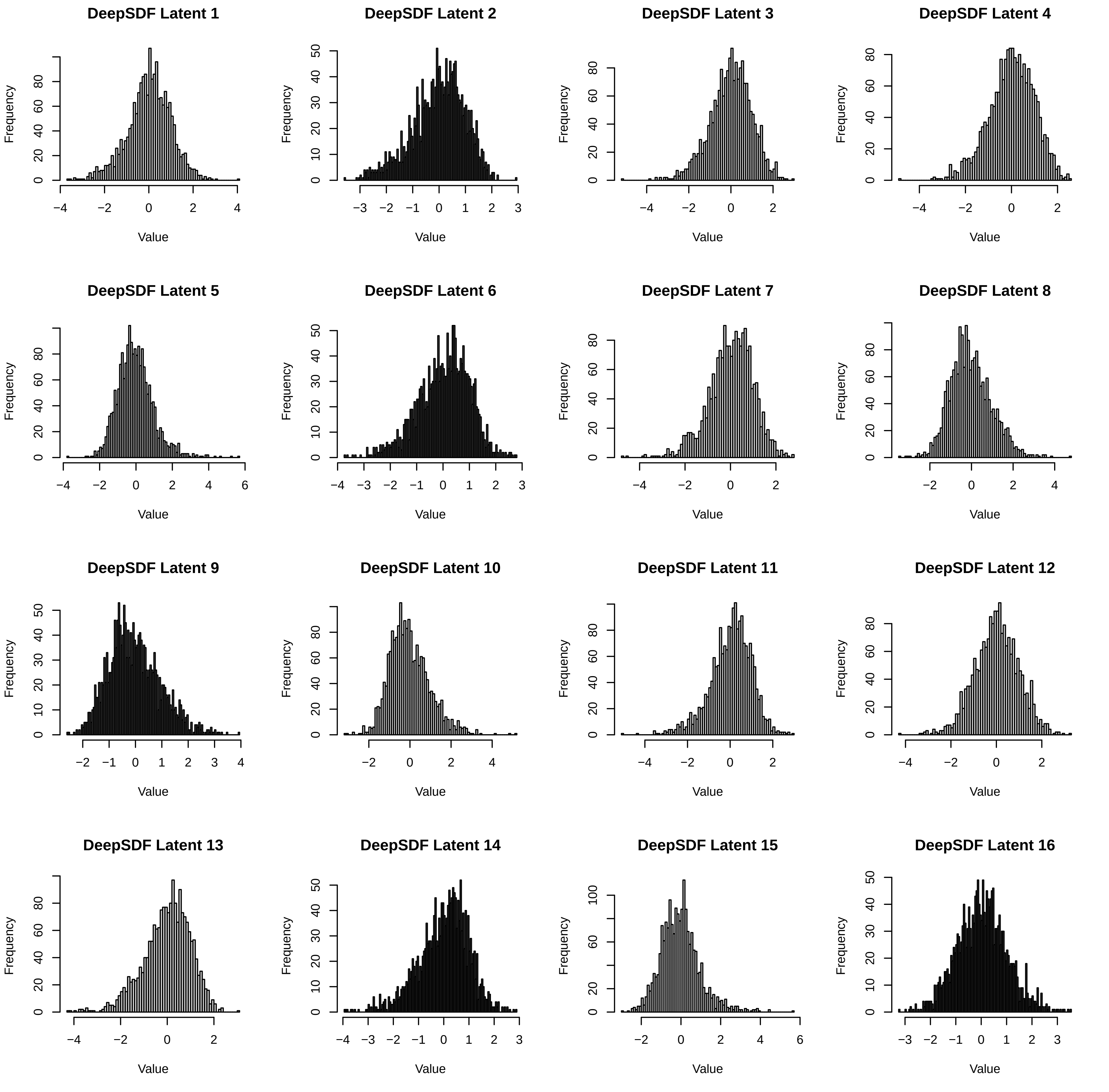
DeepSDF Latent 1
DeepSDF Latent 2
DeepSDF Latent 3
DeepSDF Latent 4
DeepSDF Latent 5
DeepSDF Latent 6
DeepSDF Latent 7
DeepSDF Latent 8
DeepSDF Latent 9
DeepSDF Latent 10
DeepSDF Latent 11
DeepSDF Latent 12
DeepSDF Latent 13
DeepSDF Latent 14
DeepSDF Latent 15
DeepSDF Latent 16
Frequency
Value

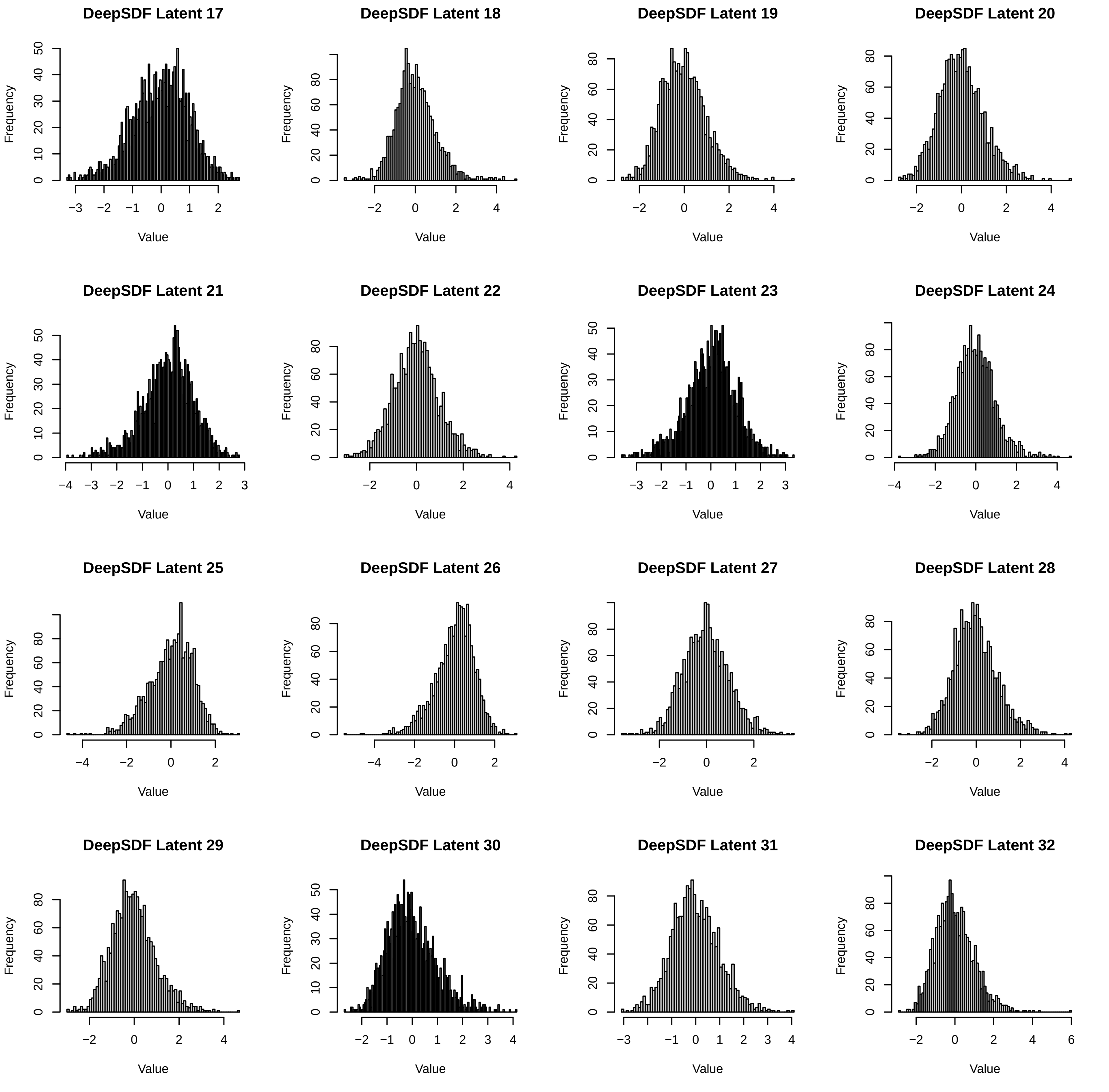
DeepSDF Latent 17
DeepSDF Latent 18
DeepSDF Latent 19
DeepSDF Latent 20
DeepSDF Latent 21
DeepSDF Latent 22
DeepSDF Latent 23
DeepSDF Latent 24
DeepSDF Latent 25
DeepSDF Latent 26
DeepSDF Latent 27
DeepSDF Latent 28
DeepSDF Latent 29
DeepSDF Latent 30
DeepSDF Latent 31
DeepSDF Latent 32
Frequency
Value

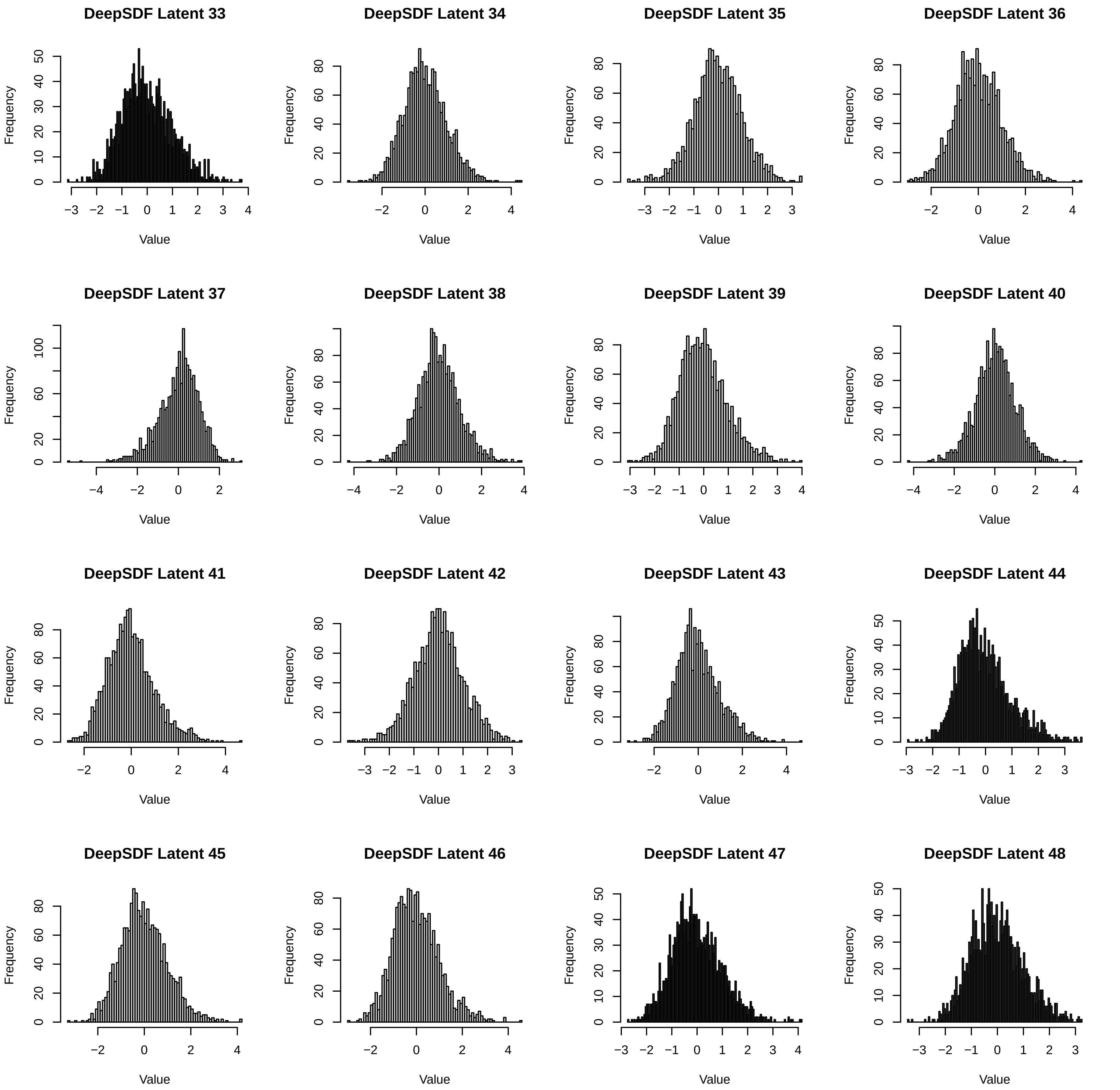
DeepSDF Latent 33
DeepSDF Latent 34
DeepSDF Latent 35
DeepSDF Latent 36
DeepSDF Latent 37
DeepSDF Latent 38
DeepSDF Latent 39
DeepSDF Latent 40
DeepSDF Latent 41
DeepSDF Latent 42
DeepSDF Latent 43
DeepSDF Latent 44
DeepSDF Latent 45
DeepSDF Latent 46
DeepSDF Latent 47
DeepSDF Latent 48
Frequency
Value

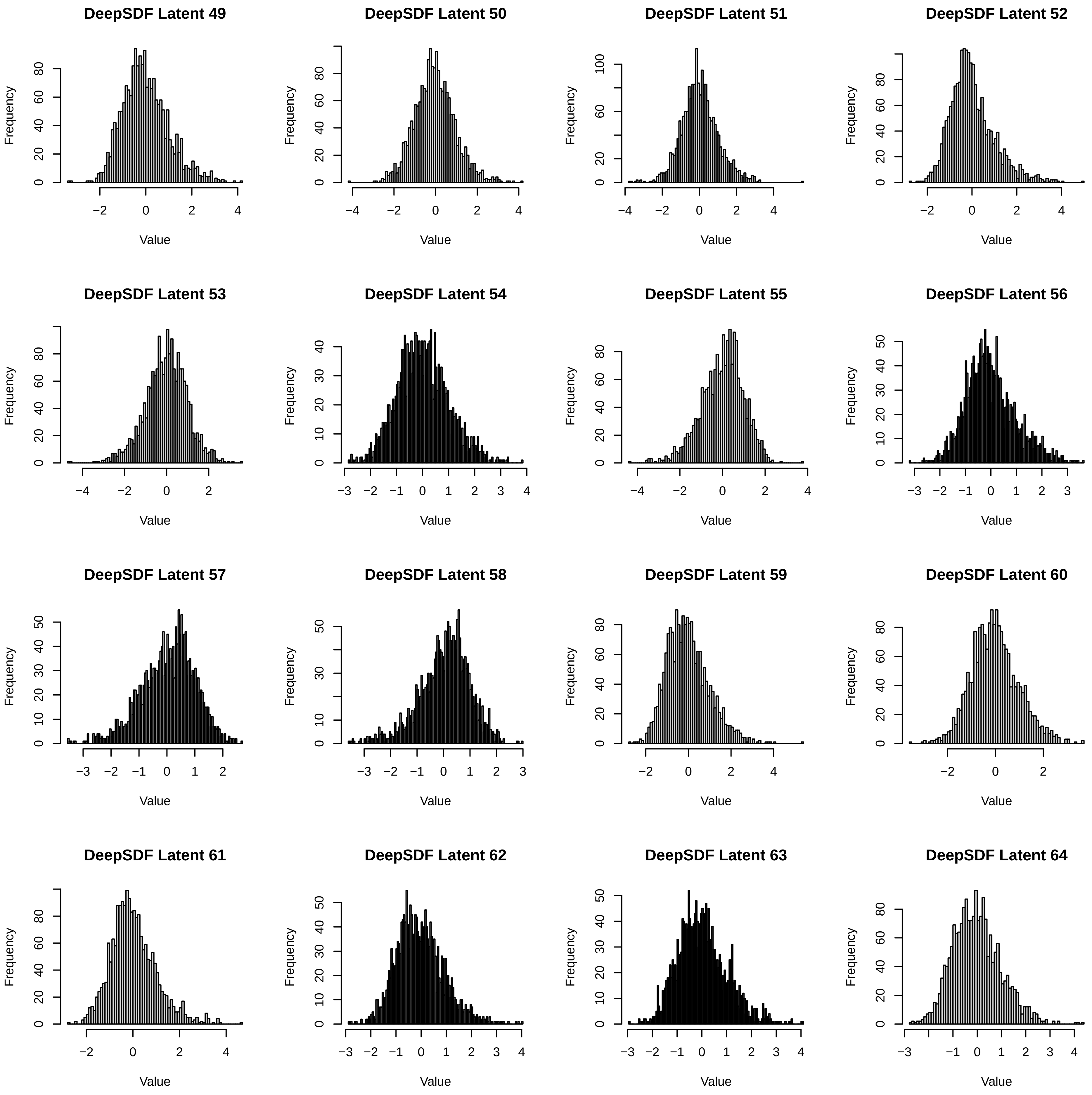
DeepSDF Latent 49
DeepSDF Latent 50
DeepSDF Latent 51
DeepSDF Latent 52
DeepSDF Latent 53
DeepSDF Latent 54
DeepSDF Latent 55
DeepSDF Latent 56
DeepSDF Latent 57
DeepSDF Latent 58
DeepSDF Latent 59
DeepSDF Latent 60
DeepSDF Latent 61
DeepSDF Latent 62
DeepSDF Latent 63
DeepSDF Latent 64
Frequency
Value

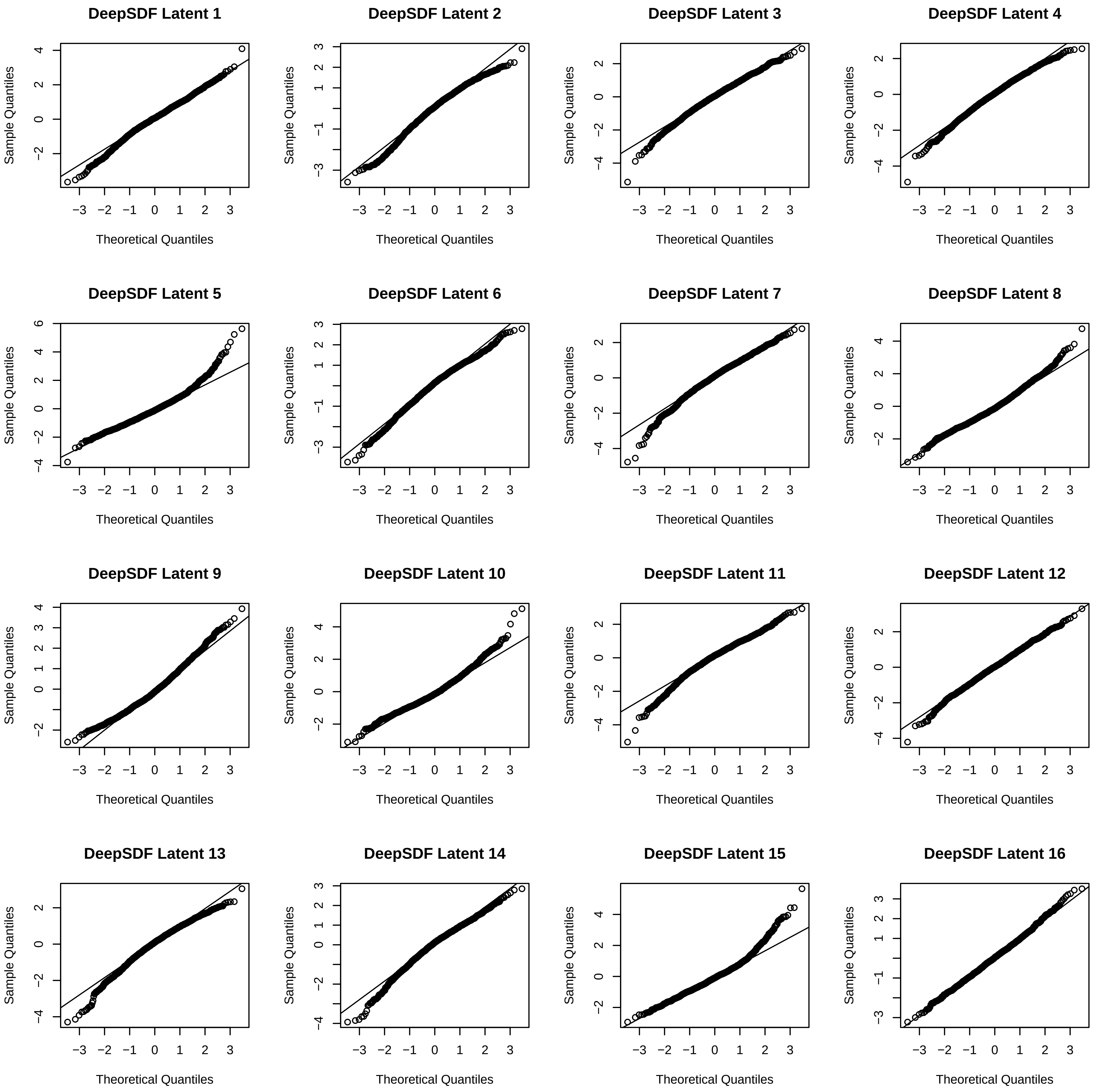
DeepSDF Latent 1
DeepSDF Latent 2
DeepSDF Latent 3
DeepSDF Latent 4
DeepSDF Latent 5
DeepSDF Latent 6
DeepSDF Latent 7
DeepSDF Latent 8
DeepSDF Latent 9
DeepSDF Latent 10
DeepSDF Latent 11
DeepSDF Latent 12
DeepSDF Latent 13
DeepSDF Latent 14
DeepSDF Latent 15
DeepSDF Latent 16
Sample Quantiles
Theoretical Quantiles

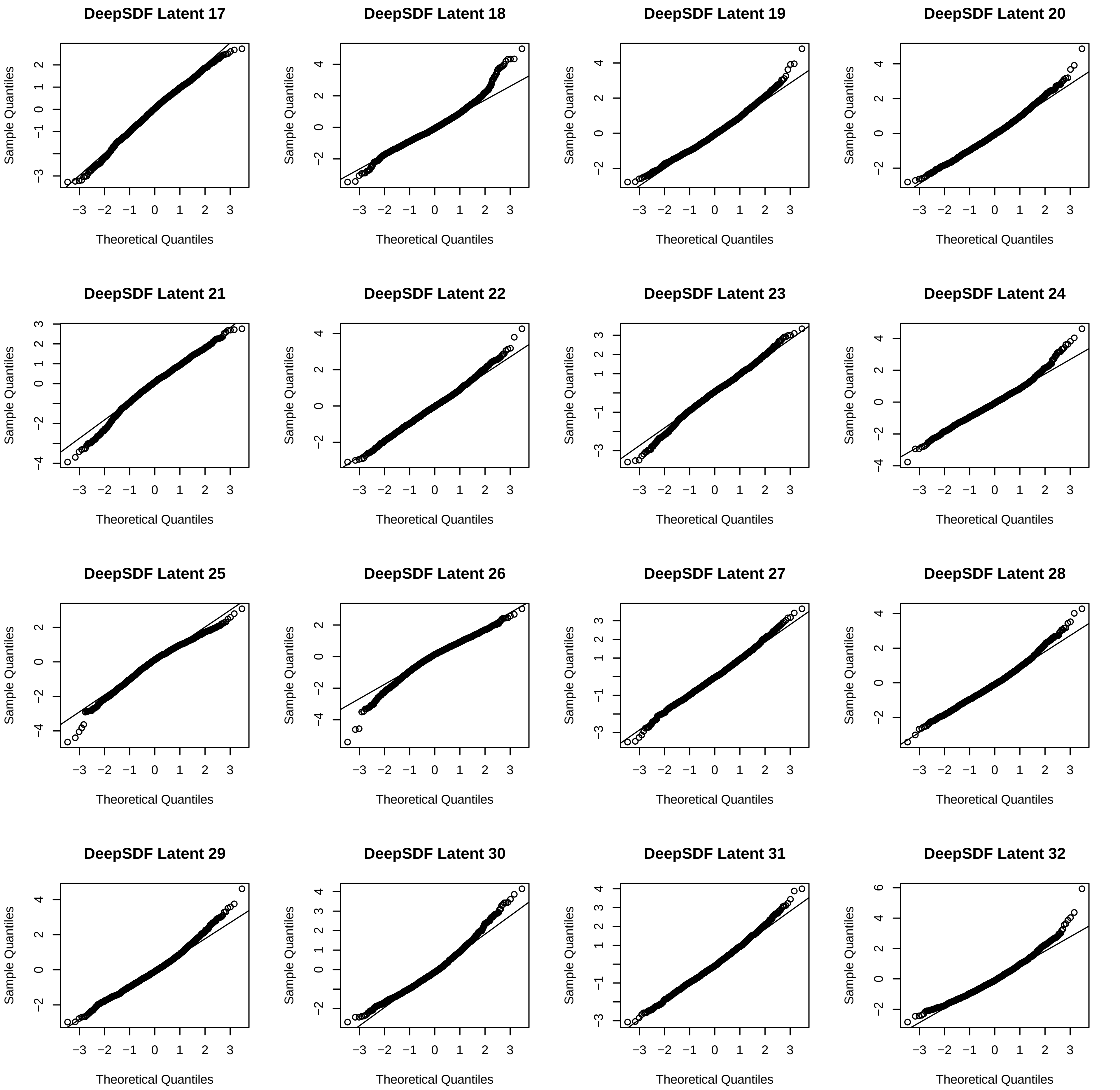
DeepSDF Latent 17
DeepSDF Latent 18
DeepSDF Latent 19
DeepSDF Latent 20
DeepSDF Latent 21
DeepSDF Latent 22
DeepSDF Latent 23
DeepSDF Latent 24
DeepSDF Latent 25
DeepSDF Latent 26
DeepSDF Latent 27
DeepSDF Latent 28
DeepSDF Latent 29
DeepSDF Latent 30
DeepSDF Latent 31
DeepSDF Latent 32
Sample Quantiles
Theoretical Quantiles

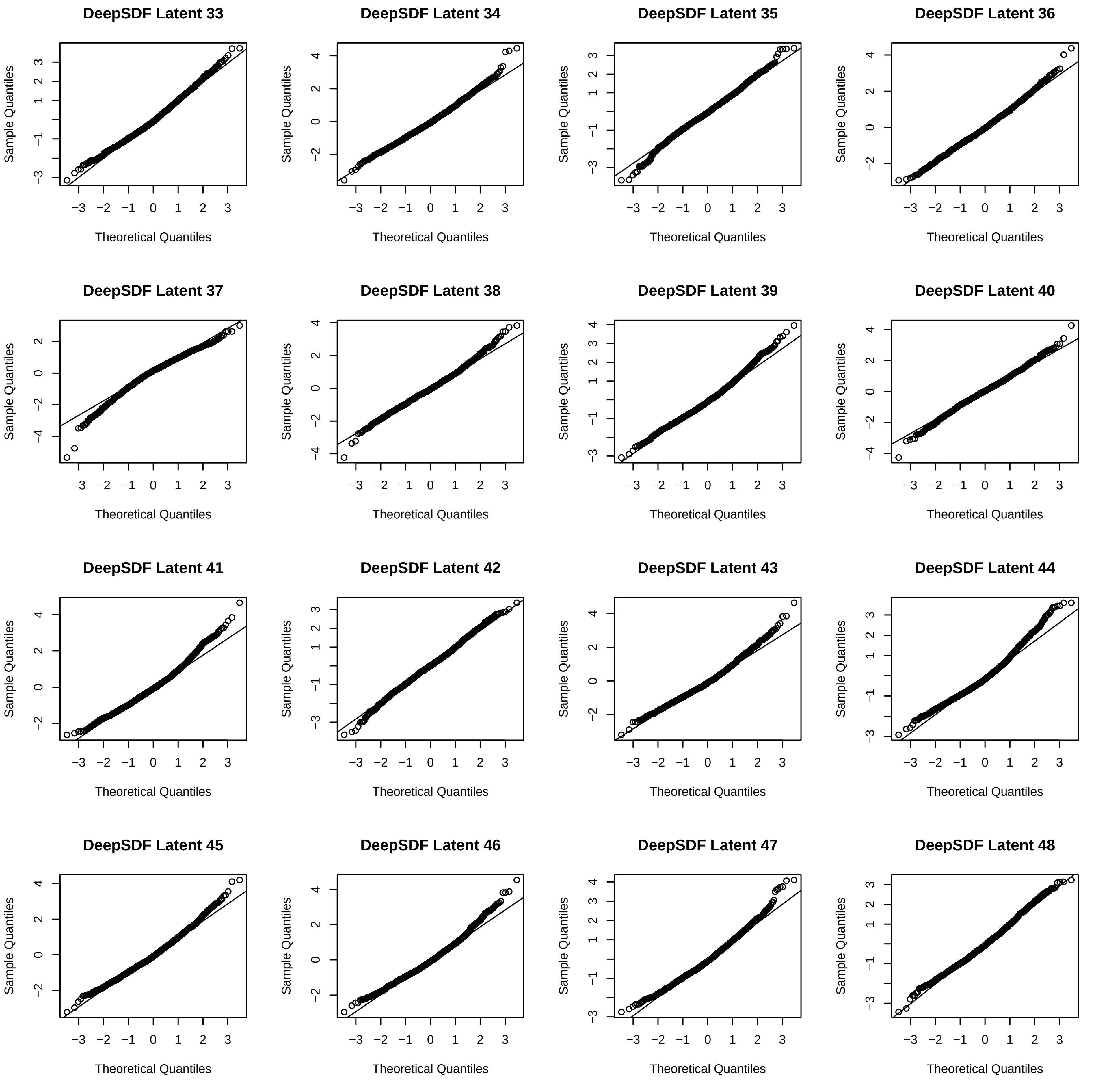
DeepSDF Latent 33
DeepSDF Latent 34
DeepSDF Latent 35
DeepSDF Latent 36
DeepSDF Latent 37
DeepSDF Latent 38
DeepSDF Latent 39
DeepSDF Latent 40
DeepSDF Latent 41
DeepSDF Latent 42
DeepSDF Latent 43
DeepSDF Latent 44
DeepSDF Latent 45
DeepSDF Latent 46
DeepSDF Latent 47
DeepSDF Latent 48
Sample Quantiles
Theoretical Quantiles

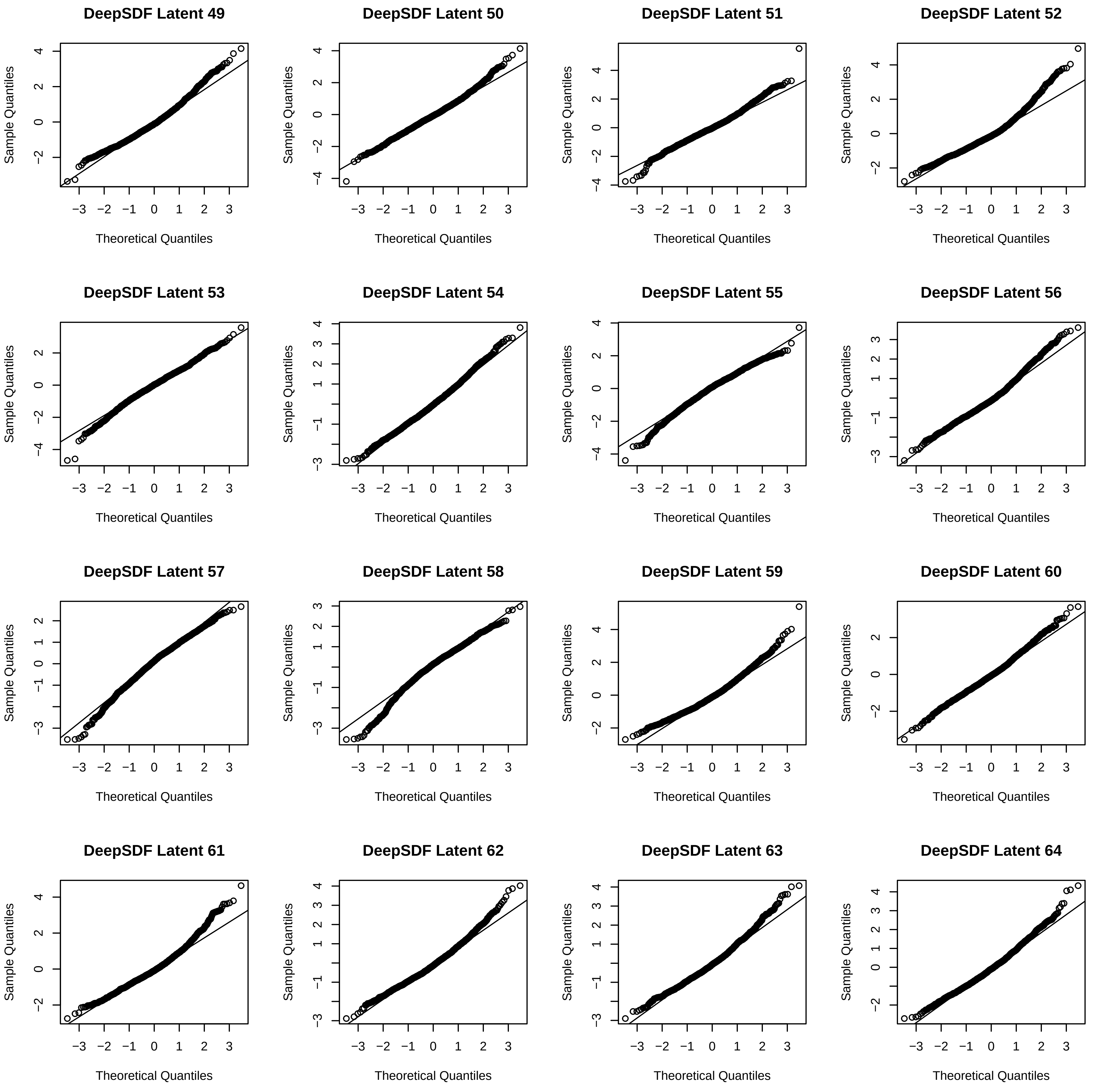
DeepSDF Latent 49
DeepSDF Latent 50
DeepSDF Latent 51
DeepSDF Latent 52
DeepSDF Latent 53
DeepSDF Latent 54
DeepSDF Latent 55
DeepSDF Latent 56
DeepSDF Latent 57
DeepSDF Latent 58
DeepSDF Latent 59
DeepSDF Latent 60
DeepSDF Latent 61
DeepSDF Latent 62
DeepSDF Latent 63
DeepSDF Latent 64
Sample Quantiles
Theoretical Quantiles

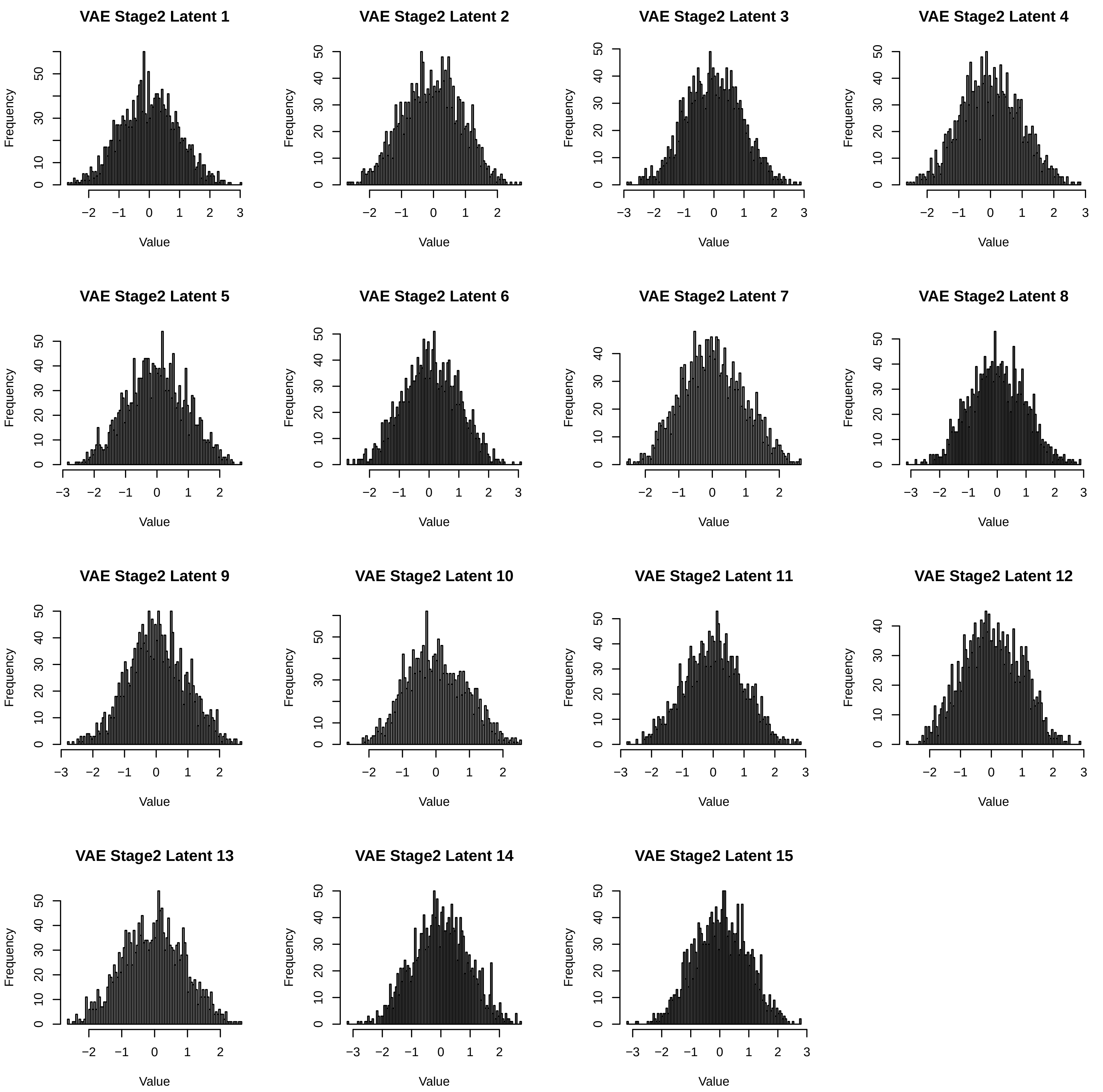
VAE Stage2 Latent 1
Frequency
Value
VAE Stage2 Latent 2
Frequency
Value
VAE Stage2 Latent 3
Frequency
Value
VAE Stage2 Latent 4
Frequency
Value
VAE Stage2 Latent 5
Frequency
Value
VAE Stage2 Latent 6
Frequency
Value
VAE Stage2 Latent 7
Frequency
Value
VAE Stage2 Latent 8
Frequency
Value
VAE Stage2 Latent 9
Frequency
Value
VAE Stage2 Latent 10
Frequency
Value
VAE Stage2 Latent 11
Frequency
Value
VAE Stage2 Latent 12
Frequency
Value
VAE Stage2 Latent 13
Frequency
Value
VAE Stage2 Latent 14
Frequency
Value
VAE Stage2 Latent 15
Frequency
Value

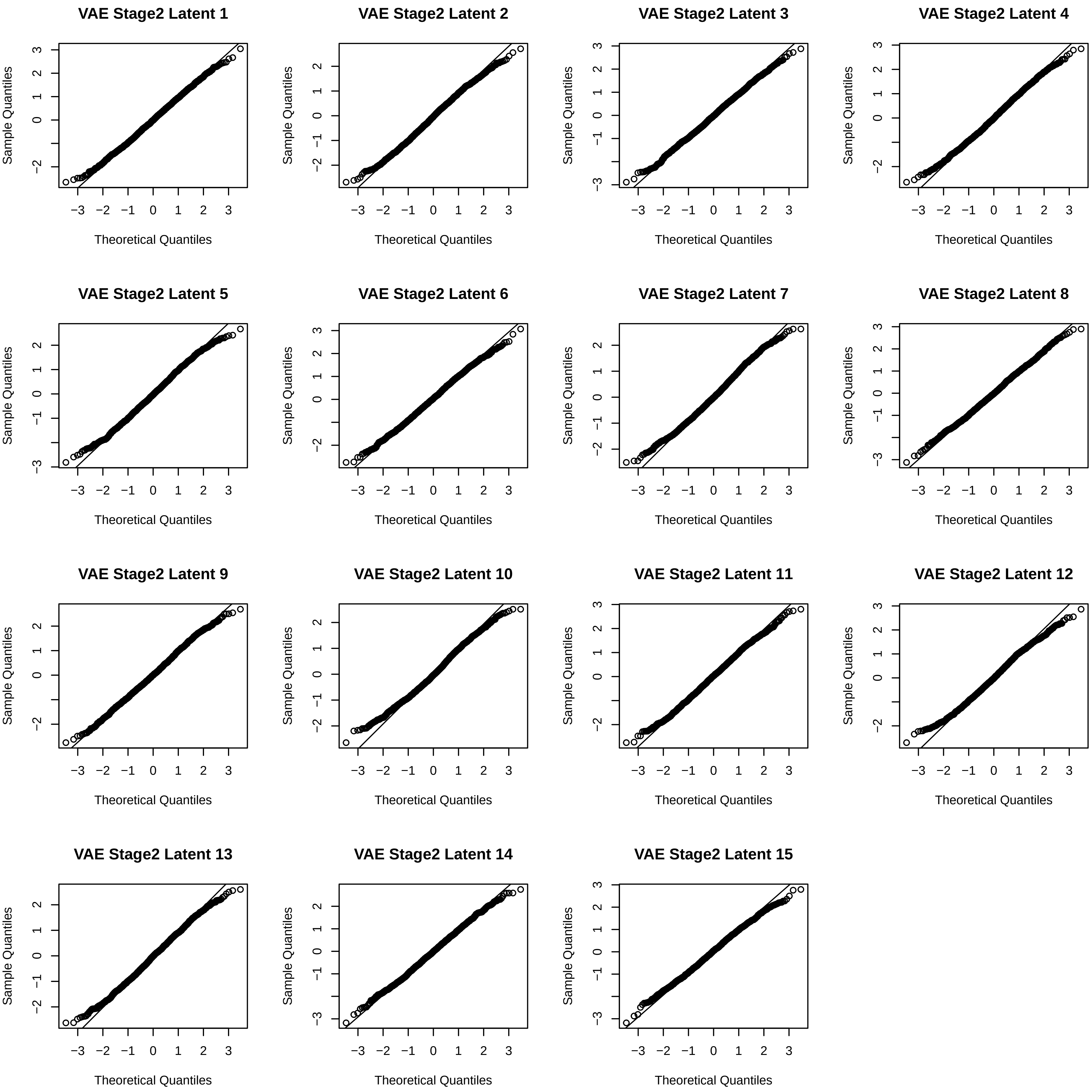
VAE Stage2 Latent 1
VAE Stage2 Latent 2
VAE Stage2 Latent 3
VAE Stage2 Latent 4
VAE Stage2 Latent 5
VAE Stage2 Latent 6
VAE Stage2 Latent 7
VAE Stage2 Latent 8
VAE Stage2 Latent 9
VAE Stage2 Latent 10
VAE Stage2 Latent 11
VAE Stage2 Latent 12
VAE Stage2 Latent 13
VAE Stage2 Latent 14
VAE Stage2 Latent 15
Sample Quantiles
Theoretical Quantiles
