# Supplementary figures and images for: Generative AI extracts ecological meaning from the complex three dimensional shapes of bird bills
Source: PLoS Comput Biol. 2025 Mar 17;21(3):e1012887. doi: 10.1371/journal.pcbi.1012887 (PMC11940575; doi:10.1371/journal.pcbi.1012887)

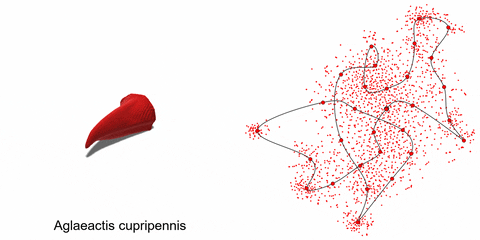

Supplement: S1 Movie — The animation shows a tour through the latent space of the model. For visualization purposes the 64 dimensional space of the model was reduced to two using t-sne on the latent codes of the observed bird beaks. Red filled circles correspond to real bird beaks observed in the dataset. The tour was constructed by choosing a set of random species from the dataset (larger red circles) and then linking them with a cubic spline (shown as a black line), in both the original 64 dimensional space and the two dimensonal t-SNE space. (GIF) [file pcbi.1012887.s002.gif]
