# Supplementary material for: Generative AI extracts ecological meaning from the complex three dimensional shapes of bird bills
Source: PLoS Comput Biol. 2025 Mar 17;21(3):e1012887. doi: 10.1371/journal.pcbi.1012887 (PMC11940575; doi:10.1371/journal.pcbi.1012887)
Supplement: Attachment — Submitted filename: Dinnage_and_Kleineberg_2024_Response_to_Reviewers.pdf [file pcbi.1012887.s005.pdf]

Thank you very much for the opportunity to submit revisions. We have written a line-by-line reply to the editor and reviewer comments below. I have highlighted editor and reviewer comments in green italics to distinguish them from our replies.

Dear Dr. Dinnage,

Thank you very much for submitting your manuscript "Generative A.I. helps extract ecological meaning from the complex three dimensional shapes of bird bills" for consideration at PLOS Computational Biology.

As with all papers reviewed by the journal, your manuscript was reviewed by members of the editorial board and by several independent reviewers. In light of the reviews (below this email), we would like to invite the resubmission of a significantly-revised version that takes into account the reviewers' comments.

I found this a very interesting study, and all three reviewers agree. However, the reviewers raised important issues which must be addressed before a decision can be made. As Reviewers #1 and #3 point out, it is important to discuss the accuracy and overall performance of the DeepSDF method on trophic niche predictions relative to more established 3D morphometric techniques. Also needing more careful discussion, as noted by Reviewer #2, is the overfitting in the trophic niche analysis suggested by the drop from 100% to 62 % between the training and test data. This feeds into the concerns raised by Reviewer #3 about the generalizability of this method to potentially much smaller datasets and other taxa.

We have done our best to address the reviewer concerns in the revisions and make detailed responses below.

We cannot make any decision about publication until we have seen the revised manuscript and your response to each of the reviewers' comments. Your revised manuscript is also likely to be sent to reviewers for further evaluation.

When you are ready to resubmit, please upload the following:

[1] A letter containing a detailed list of your responses to the review comments and a description of the changes you have made in the manuscript. Please note while forming your response, if your article is accepted, you may have the opportunity to make the peer review history publicly available. The record will include editor decision letters (with reviews) and your responses to reviewer comments. If eligible, we will contact you to opt in or out.

We have included this below.

[2] Two versions of the revised manuscript: one with either highlights or tracked changes denoting where the text has been changed; the other a clean version (uploaded as the manuscript file).

We apologize, but do not know how to do this since we wrote the manuscript and revisions using the Authorea web app, which does not have track changes functionality. We have changed a lot of the text so highlighting all changes would be an enormous amount of work and take a feat of memory I am not sure I have in me. As an alternative I exported a word document of the manuscript before I started editing, and after. I then used Microsoft Word's 'compare documents' feature to create something like a track changes document. However the main manuscript is still currently a PDF.

I*mportant additional instructions are given below your reviewer comments.*

Please prepare and submit your revised manuscript within 60 days. If you anticipate any delay, please let us know the expected resubmission date by replying to this email. Please note that revised manuscripts received after the 60-day due date may require evaluation and peer review similar to newly submitted manuscripts.

Thank you again for your submission. We hope that our editorial process has been constructive so far, and we welcome your feedback at any time. Please don't hesitate to contact us if you have any questions or comments.

Sincerely,

Rafael D'Andrea, Ph.D.
Academic Editor
PLOS Computational Biology

Feilim Mac Gabhann
Editor-in-Chief
PLOS Computational Biology

****

Reviewer's Responses to Questions

Comments to the Authors:
Please note here if the review is uploaded as an attachment.

Reviewer #1: This paper reports on an AI-based approach for analysing complex shape variation in 3-d scans of bird beaks. The study employs an 'autodecoder' architecture combined with a subsequent variational autoencoder (VAE) step to first learn and then generate coherent representations of bill shape variation. The authors go on to show that the latent vectors learnt by the model capture intuitive aspects of bill shape variation ('elongation', 'broadness') and can be used to predict variation in species feeding ecology with some accuracy.

Overall I think this is a valuable study that describes a potentially important new approach for extracting meaningful representations of complex 3-d morphological datasets such as this. The manuscript is generally clearly written and well-presented and the visualisations are useful and impressive. That said, from the perspective of a comparative biologist with experience of working with 3-d bill shape data from museum specimens I have some queries/suggestions/concerns to consider:

Thank you very much for your very helpful comments. We hope we have managed to address most of your concerns, though I don't believe we have been able to solve everything you bring up, though we did our best in the time we had. Beyond this manuscript you have given us a lot to think about as we move forward in developing this and related methods further, and we appreciate it.

1. As mentioned, from experience I am aware that the positioning of the lower bill relative to the upper bill can vary idiosyncratically bird museum specimens as a result of preservation style, damage etc. The method here uses the whole bill scan as input and therefore this unhelpful variation in relative bill position is presumably encoded into the latent vector representation and may be problematic. Are the authors able to probe the extent to which variation in the relative positioning of the upper and lower beak is encoded by the model and whether this is problematic for downstream analysis? For example, could the model be retrained with only the top bill

included as input and the results compared? I raise this as the original study to analyse the beak shape data (Cooney et al. 2017, Nature) focused only on the shape of the upper bill to avoid this issue.

Thank you very much for your thoughts on this. I had only briefly considered this issue in the original study, but you are correct, this could in some cases cause problems for the overall analysis. One advantage of the approach here however is that even if some beaks have very strange or misaligned lower sections, this will just cause them to appear in a low density region of the latent space, but they will not affect the positioning of other species. Therefore it is possible to exclude these specimens post-hoc without having rerun the generative AI model. This is what I have done in the revised manuscript. I have gone through all the original beak meshes and identified any that had significant misalignment of the upper and lower jaw (and also any other issues that created an unusual mesh not reflective of the beak shape). This resulted in excluding 89 beaks from the subsequent analysis. I have added a discussion of this to the manuscript. Your suggestion of only analysing the top of the beak is interesting but very challenging in this context, because this would require slicing the 3d meshes in a particular way, and then the hole created would have to be sealed. This would have to be done manually. So in this way, the landmark based approach of Cooney et al 2017 would have an advantage. However, it is arguable that excluding 89 out of 2021 meshes entirely is preferable to removing an entire half of the beak's full form for all the beaks.

2. Regarding the latent vector discovery analysis (starting line 304), as well as the AVONET measurements, why not also use the principal component (PC) scores of 3-d bill shape generated in the original paper (Cooney et al. 2017, Nature) to probe for meaningful vectors? The Cooney et al. PC scores have the benefit of being measured on the precisely the same dataset as used by the present study and therefore should be more comparable (though less independent) than the AVONET linear measurements. These PC scores also capture more complex shape variation than is possible from the more simple length, width depth measurements, such as 'degree of hookiness' alluded to in the Discussion (line 538).

We think this is an excellent suggestion. I would like to pursue this for a follow-up analysis, but I think it falls a little outside the scope of this paper. I am concerned about the lack of independence, especially since I am now using the PC scores from Cooney et al in several other places in the manuscript.

3. I think it the discussion of the tropic niche results needs to be extensively revised to incorporate the findings of Pigot et al. (2020) (https://doi.org/10.1038/s41559-019-1070-4), who performed a very similar classification analysis using not only beak shape data but also other morphological measurements (e.g. leg morphology) as proposed in the Discussion (line 471). Currently Pigot et al. (2020) is not cited and is conspicuous by its absence.

Indeed it was an oversight to not cite and discuss Pigot et al (2020). I am not sure how I missed this, but thank you for pointing it out. We have now added a discussion and comparison of this paper to our discussion. Our results seem to be very consistent with theirs.

4. Regarding the trophic niche analysis, in my view it would be highly beneficial to compare the prediction accuracy of the latent vectors generated here to that based on other beak shape datasets. In particular, it would be informative to contrast the performance of the latent vectors against the predictive accuracy of (i) the PCs scores from the original paper (Cooney et al. 2017) and (ii) the linear beak shape measurements from AVONET (or Pigot et al. 2020). Currently, the authors argue that their latent vector representations are 'ecologically meaningful', but it's not clear how meaningful they are relative to alternative more established techniques based on linear measurements and 3-d morphometrics.

Thank you for the suggestion. We have added a comparative analysis to the PC scores used in Cooney et al. We found that the DeepSDF based latent variables were not particularly better at distinguishing trophic niches than PC scores. On further reflection this is perhaps not surprising. It seems the biggest barrier to effectively separate trophic niches is in classifying omnivores, but this is perhaps an impossible task, regardless of the sophistication of morphology representations, since omnivores are somewhat an artificial category. They really should be regarded as transitional between other more specialized diets. However, we did discover interestingly that a linear classifier was more effective using DeepSDF latent variables as compared to PC scores.

Minor:

L299 – what parameter settings were used to generate the t-SNE visualisation?

We added parameter settings to the manuscript.

L329 – what parameter settings were used to generate the UMAP visualisation?

We added parameter settings to the manuscript.

Figure 4 – It would be useful perhaps to add an additional panel to this figure with datapoints coloured by taxonomic group (e.g. order). This would help provide insight into the composition of 'outlier' groups (e.g. points in the top left of fig. 4a), specifically whether they constitute sets of related species or independently evolved convergent morphologies.

We added direct labels describing the dominant taxonomic groups in the outlier clusters, out of interest. We tried a figure coloured by order but there were too many orders to be able to distinguish their colours.

Reviewer #2:

In their manuscript, "Generative A.I. helps extract ecological meaning from the complex three dimensional shapes of bird bills", Dinnage & Kleineberg train a deep representational learning model on 2000+ 3D scans of bird beaks from the public MarkMyBird dataset. The learned latent space is further associated with measures of beak elongation and broadness, showing that ecologically relevant morphological variation is represented by the learned latent space. The model is also able to predict the trophic niche associated with a particular beak shape.

The study represents an interesting and novel application of generative A.I. models to the study of 3D morphological shape and correlations with ecological roles. In general, the manuscript is also well-written and clear, though there are some sections that would benefit from more explicit quantification (see below).

Thank you very much for your praise and helpful comments. We hope we have addressed the majority of your concerns.

The most major issue I identified is that it appears that the trophic niche prediction model is overfitted, given the very high accuracy (100%) on the training set but comparatively poor performance on the test set (64%). This suggests that the model has basically memorized the training set but has quite poor generalization capabilities. While the authors make some arguments as to why the results are valid despite the relatively low test accuracy, they never explicitly address this problem of overfitting in the Results or Discussion (the word 'overfitting' is only mentioned with respect to regularization in the Methods [line 287], and only in a general sense). This omission needs to be corrected as the model, based on what is presented, is very clearly

overfitted and this is a fundamental issue in these types of learning problems. What, if anything, was done to try to fix the overfitting problem? Is the model too complex, are there too many learning parameters, is there not enough data, etc.? Some of these questions are answered (or implied) in other parts of the paper, but I think there needs to be a focused discussion with specific reference to the fact that the model is overfitted.

We did not think it needed addressing because this is a very common phenomenon in classification models. The model is overfit, but we did all we could to prevent it by tuning the hyper-parameters to maximize performance on held-out validation data. Now that we have compared our original model to one using PCA scores we can say that it is not just the DeepSDF latent variables, the PCA model also had 100% accuracy on the training data. This is sometimes unavoidable, but as long as we are only evaluating the model on a held-out test dataset then this is fair. I think the problem is that we reported the accuracy on the training dataset but I don't think this is common practice. For example, Pigot et al (2020), which reviewer 1 pointed out, did a similar random forest model to predict trophic niche and they did not report performance on the training data. Because of this, we no longer report accuracy on the training data in order to avoid a potential distraction trying to understand why the model is so overfit. To be honest we are also a little surprised that it is so extremely overfit, but we have not been able to do any better no matter how much we tune the model's hyper-parameters. As we discuss now in the paper, this level of accuracy is quite similar to previously reported accuracy levels for this sort of problem. Our balance accuracy is actually quite good at 88%, this takes into account data imbalance. We also discuss why this discrepancy happens in our revisions, that much of the low accuracy overall is because of poor prediction of Omnivores, which make up a fair bit of the dataset. This is probably unavoidable, since omnivores naturally have generalized morphology.

With regard to Figure 5, I think it would help interpretability if: (1) The # of samples per class is marked for each trophic niche (on both the left and the right). The current "freq" axis is not very useful because it is spread across all classes; (2) The % match is shown on the left side of the plot -- for example, 80% match in prediction for Invertivore class, etc. These numbers should also be folded into the discussion in the text (lines 392-406), which is too qualitative at the moment, relying on ambiguous qualifiers such as "most", "more", "sometimes", etc. The class labels should also be out to the side instead of overlapping the flows for the sake of visibility.

We changed figure 5 to a more standard confusion matrix figure, directly plotting the proportion of correctly classified observations in each trophic class. We have added the sample sizes of each trophic niche to a table (Table 1)

Minor Comments

Line 128-131: This sentence is confusingly constructed and hard to understand; consider rewriting as multiple sentences.

We reworded.

Line 198: "conditional" not "confitional"

Fixed.

I think it would make sense to put the section describing VAEs (lines 270-293) earlier than where it is now, as currently the manuscript discusses VAEs before you define what they are. Towards the beginning of the Trophic Niche Conditional VAE section would probably be best (rather than at the end, as now).

We have reordered this section as suggested.

Line 348: "well known" not "well know"

Fixed.

The authors should provide a table or list of the number of samples per trophic niche when discussing the class imbalance issue (lines 347-357).

Done.

Line 358: There is an extra space after "min_n parameters"

Fixed.

Line 385: "trophic" not "tropich"

Fixed.

Line 397-400: This is a run-on sentence.

Doesn't seem too run-on to me.

Reviewer #3: *The review was uploaded as an attachment.*

<u>Comments to the Corresponding Author</u>

This manuscript employs a deep generative modelling approach called DeepSDF to visualize and analyze beak shape variation across different species. The authors additionally show how the latent morphology data can be used in various downstream analyses, such as classification. The main contribution of this work is the application of a slightly modified DeepSDF method to generate morphological data. The authors show via dimensionality reduction methods that the latent data result in clustering of trophic niches. They also report that their random forest classification model was able to correctly predict the trophic niche of most birds. The figures summarized these tests well and were expertly put together.

Our thanks. We are glad you liked the figures. And thank you for your comments for improving the manuscript. We hope we have addressed the majority of your expressed concerns in our revisions.

While the modeling framework and visualizations were interesting, I have several major concerns. I was not actually sure what the novelty or overall objective of this paper was. On the one hand, the manuscript comes across as attempting to introduce a refined DeepSDF method, although most of the math and formulae shared throughout the work are taken from the original paper. If there was a methodological angle, a lot more needed to be presented. For example, how does this method perform on different sample sizes, or maybe different organisms, as opposed to a (relatively large) sample of 2000 bird beak meshes? And where are the loss curves or discussions of reconstruction error? On the other hand, it is trying to argue that the learned latent data have increased ecological meaning, but this is not explicitly tested relative to another method. As someone unfamiliar with bird anatomy and their classification accuracies, it was not clear to me whether this approach actually outperforms others. In other words, the findings do not actually support the claims.

We never intended to suggest we were refining the DeepSDF method. We only intended to introduce the method to ecologists and other organismal biologists as a potentially promising method. We also never claimed

the method outperforms others, only that it performs well for a relevant task that ecologists are interested in. The goal of the paper was to introduce the method and some of its potential, hoping in particular to attract interest in furthering the development of this type of method in general. We now have tested the method against using simpler PCA based variables from landmarks (as suggested by Reviewer 1), and found our method does not do better, but it does do nearly as well, which we think is enough to continue development and further application of the model, considering its greater potential. I hope we don't get trapped into the cycle of state of the art (SOTA) models at all cost that has taken hold in some fields of machine learning (and bioinformatics). We have added a section attempting to address your comments about smaller sample size datasets. Specifically we believe the method will work on datasets as small as a few hundred, based on experience, but that a better approach to dealing with small curated datasets would be to train a large model on a large amount of data, and then fine-tune this large model on the smaller dataset. This is assuming a large dataset is available for the data of interest, but we see this as where we are eventually heading with this type of analysis, that is, towards training large 'foundation models' that can be reused and refined.

The other issue I had was the overall presentation of the manuscript. There were a number of spelling and punctuation issues, the writing was not concise, and it was not organized according to the journal's policies. These problems can obviously be fixed, but as is, they greatly hindered the reading.

We have fixed all spelling and punctuation errors and have reorganized the manuscript according to the journal's policies.

Minor comments

Lines 44-60: Talking about development provides a set of instructions to assemble a phenotype through non-linear probabilistic dynamics; development as a non-linear decoder. Strong argument.

Thank you!

Line 61: A.I. acronym should be artificial intelligence (AI).

Fixed.

Line 73: three-dimensional (3D).

Fixed.

Line 75: I agree with these challenges.

We are glad you agree!

Line 80: 3D not 3d.

Fixed.

Line 100-102: demonstrate the utility of deep generative models? What does that mean?

We have revised this section. We now say we demonstrate that the method captures ecological 'signal' at least as well as previous simpler methods. This demonstrates that the method can now begin to be integrated into the field, allowing organismal biologists to take advantage of its other advantages, e.g. reusability of the trained model, ability to easily generate data for validation or various other tasks.

Lines 103-112: This paragraph comes out of nowhere and should be better integrated into the introduction. Details of the dataset should be provided below in the Materials and Methods section.

We think the dataset is important to be introduced in the introduction, since it is key to the study overall. Though the method of course is general, it has been strongly informed by the details of the dataset and the types of questions that can be asked with it.

Line 113: Results should follow the Introduction, as per the journal guidelines, correct?

The sections have been rearranged.

Lines 115-117: Even though the dataset is publicly available, there should at least be some details on the specific scanner, how it acquires images, the surface reconstruction, etc.

We disagree. This doesn't seem relevant to the study, since in theory the method can be used on any 3D data scanned using any method, including 3D photogrammetry. Those interested can easily look this up from the referenced paper and website.

Line 119: The authors don't clarify what packages, etc. were used in the "algorithm written in R" until line 142. Either state your completed the following steps: "…" etc., or clarify what packages were used upfront.

We moved the package references up front.

Lines 123-131: This reorientation step, where the authors use existing landmarks to align the data, is not as trivial as the authors suggest. Landmark-free (i.e., data-driven surface or volumetric registration) methods are often error-prone and difficult to implement.

We were not trying to make it sound trivial, just that an option exists if landmarks are not available. We also clarify here that we only needed three landmarks for orientation, much less than the several dozen required for the original geometric morphometrics analysis (which also has to align but uses a much more complicated Procrustes analysis for alignment).

Line 128: Extra spacing? And this website is repeated too much.

Fixed.

Line 145: Again, 3D, not 3 dimensional.

Fixed.

Line 152-153: Confusing wording.

We are not sure what is confusing about it?

Lines 167-187 (Model Specifics): The math is important but it seems overkill here, especially on pg. 8, as the authors are not introducing a new method. They are simply restating formulae from the original Park et al. (2019) signed distance function paper.

We actually rederived the MAP estimate from first principles, for our own and our reader's benefit, and our derivation is more detailed than that in Park et al. (2019). Computer science folks may be very familiar with this derivation but we don't believe most biologists are, so we think this is not unreasonable to include, especially now that the manuscript has been rearranged so that this section appears closer to the end of the paper. It

perhaps could be moved to the Supplement however if it seems too heavy on the math. We will leave it to the editor's discretion to request moving it.

Line 177: 3D instead of 3d. Nitpicking, but this should be consistent throughout the paper.

Fixed.

Line 181: Why was δ set to 0.1? Was this tested empirically?

This was used in the original Park et al. (2019) paper. We did not test it empirically in our case as this default seems to work well for many datasets in our experience. Park et al. (2019) did not find that it had a large effect in their sensitivity analyses.

Line 189: Wording? "Note that using"?

Fixed.

Line 191: Contraction.

Fixed.

Line 196: Spacing.

Fixed.

Line 200: I would argue a dataset of 2,000 bird beaks is very large for a biological study, not relatively small.

You are correct, it is quite large for a biological study, but small for a typical machine learning dataset (for generative AI methods at least). We have expanded this point a little to be more clear what we are getting at.

Line 202-203: Introduce VAE acronym?

Done.

Line 215: Spacing.

Fixed.

Lines 205-213: They use a modified neural net that appears to improve performance in Park et al. (2019).

I'm afraid I don't understand what is meant by this comment.

Line 215: Spacing.

Fixed.

Lines 214-225: Training the DeepSDF model. Do they have any figures of the loss?

Unfortunately, though the loss data was collected during training the files containing this data were lost during a challenging move between Australia and the United States by the first author. I have only managed to find the loss values for the final short 50 epoch training run of the model (the model was trained in several runs, each time resuming from the previous training run, and the previous run's losses have been lost). This shows the expected decrease followed by a plateau showing the model was reaching an asymptote (though loss was still

decreasing suggesting the model could still be improved with longer training). I am reproducing this data here, because it doesn't seem quite suitable to include in the manuscript, since it is incomplete. We have now explained in the manuscript that the loss values are not that informative of the fit of the model, since it is only the accuracy around the zero isosurface that really affects the 3d shape, but the loss includes the accuracy of the entire signed distance field. So the fit has to be evaluated more informally by eye. We have included a link to a Supporting File on Figshare that has all original beak meshes plotted side by side with their reconstruction from our model, to support our claim that the model fits well. If the reviewers feel strongly that a loss curve is necessary for the publication then we will have to retrain the model and recollect this data. We are willing to do this but it seems a bit of an unfortunate waste of computation (though I take full responsibility for my lack of good data management that led to the situation).

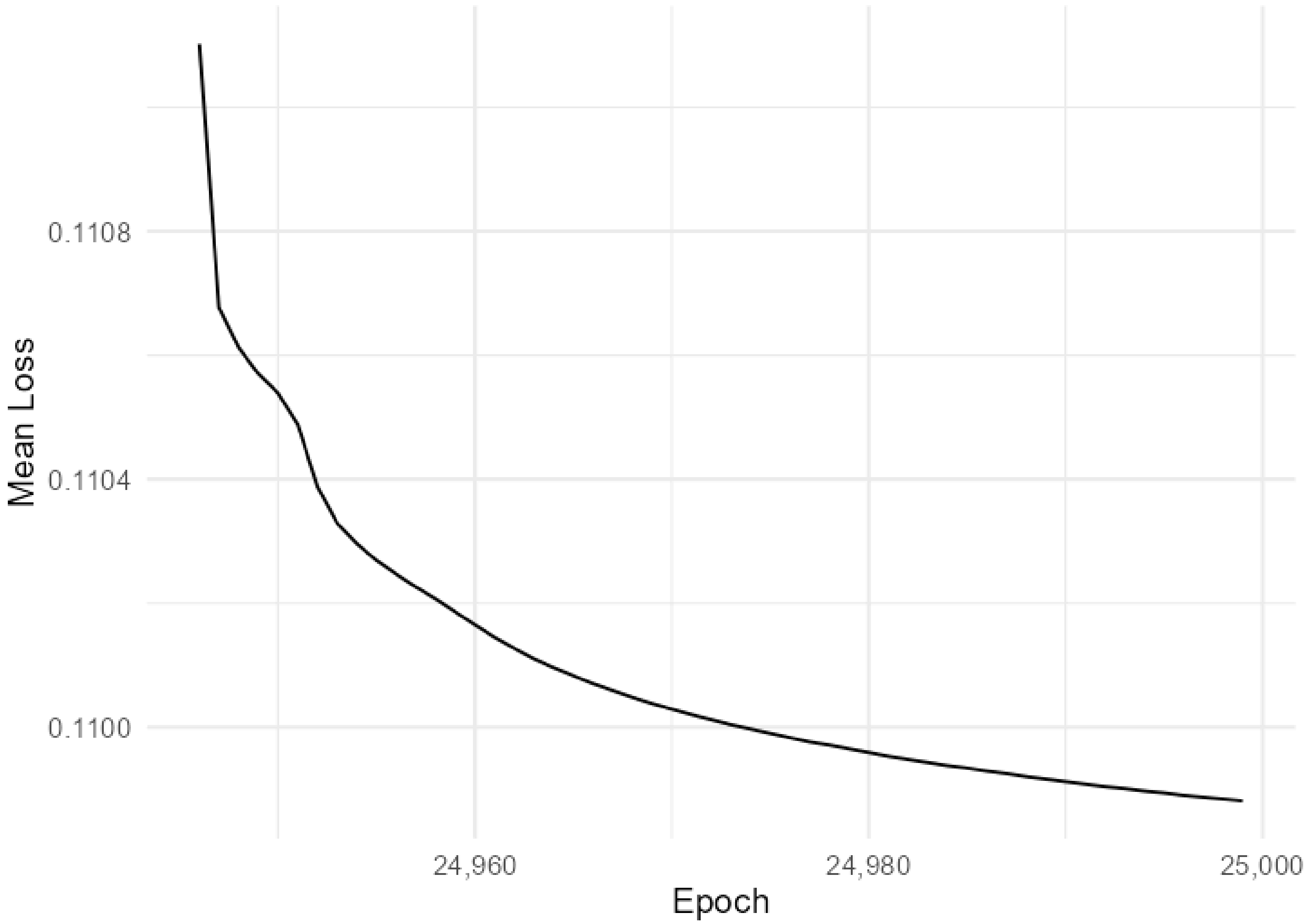

Lines 234-243: DeepSDF implemented in PyTorch for Python; model has another R implementation using the torch package? Bouncing between R and Python.

We don't see this as a problem. The R implementation is identical to the Python one, we have just essentially imported the Python model into R to make downstream analysis and visualization easier, and also make it more accessible to many organismal biologists who tend to use R more than Python (e.g. for geometric morphometrics). Incidentally the R model can now be retrained or fine-tuned in R if desired. This seems advantageous rather than problematic.

Line 256: "in our case"

Fixed

Line 259: Spacing.

Fixed.

Lines 259 to 261: Talking about two-stage VAEs and fitting a second-stage VAE to the first stage DeepSDF latent vector data, then they say the second-stage VAE estimated latent space conformed strongly to an independent Gaussian distribution, whereas the first latent vectors deviated substantially? Where are the results/figures?

We added figures showing this to the Supplementary Material.

Lines 270-287: They start discussing VAEs at length here. Why not do this at the beginning of the section?

It has been moved to the beginning of the section, as suggested.

Lines 297-298: Wording? "Using common machine learning algorithms"?

Fixed.

Line 346: Wording? "Were"?

Fixed.

Line 348: Wording? "Well-known"?

Fixed

Lines 353-356: Why not just upsample or downsample? Why use a combination? Were you trying to land on a specific number of observations per class?

This was actually an error in the original submission. In fact we only upsampled. In the revised manuscript we actually used our conditional VAE to generate synthetic data for the upsampling.

Line 358: Punctuation.

Fixed.

Line 365: Wording? "Revealed"?

Fixed.

Line 367: What downstream tasks?

Line removed.

Line 368: Is there any discussion of reconstruction error anywhere? I don't see it in Fig. 1.

Reconstruction error needs to be evaluated qualitatively because the overall error of the SDF is not too informative since an accurate shape is mostly determined by high accuracy close to the zero isosurface and error further away has less effect. We added the final loss achieved and also some more qualitative description of the model reconstruction quality. We also include images of all 2021 beak reconstructions along with their original meshes as Supplementary material.

Lines 373 to 382: I don't understand why there needs to be a discussion about 'entanglement'. The authors are basically just hinting at morphological integration, or the tendency for structures to covary. This is a ubiquitous feature of organisms due to shared developmental pathways, so one would actually expect (and hope to see) how features are 'entangled'.

One of the goals of representation learning is to find good representations. Some have suggested that a desirable characteristic of a representation is that it 'disentangles' meaningful axes of variation. This is more about the latent space, and not the 'morphological' space, which, as you correctly point out, should be 'entangled'. We've modified this section to make this clear as we agree the original passage was a bit confusing on this point.

Lines 386-388: I would like to see how UMAP compares to standard linear dimensionality reduction methods (e.g., PCA/CVA and PLS) with these data.

Linear dimension reduction techniques like PCA are inappropriate for latent space variables because in general the latent space of generative models are non-euclidean. UMAP is designed to take into account that the data may lie on non-linear manifold (though technically it still assumes euclidean geometry but provides a better approximation since it connects data points that are close together in a graph, where euclidean distance is a better approximation of a non-euclidean geodesic).

Arvanitidis, G., Hansen, L.K., & Hauberg, S. (2017). Latent Space Oddity: on the Curvature of Deep Generative Models. *arXiv: Machine Learning*.

Shao, H., Kumar, A., & Fletcher, P.T. (2017). The Riemannian Geometry of Deep Generative Models. *2018 IEEE/CVF Conference on Computer Vision and Pattern Recognition Workshops (CVPRW)*, 428-4288.

Lines 392-406: It's interesting to see these classification results, but there's no comparison. For example, what classification accuracies would you get with linear measurements, landmarks, etc.?

I have now made this comparison with PCA based variables.

Line 405: Just say most broad or fat-tailed instead of most 'diffuse'? Odd phrasing.

Done.

Lines 427-447: This is far too lengthy. Classification is not the main point of this paper. Suggesting one or two likely reasons for omnivore misclassification seems sufficient.

This section has been shortened.

Have the authors made all data and (if applicable) computational code underlying the findings in their manuscript fully available?

The PLOS Data policy requires authors to make all data and code underlying the findings described in their manuscript fully available without restriction, with rare exception (please refer to the Data Availability Statement in the manuscript PDF file). The data and code should be provided as part of the manuscript or its supporting information, or deposited to a public repository. For example, in addition to summary statistics, the data points behind means, medians and variance measures should be available. If there are restrictions on publicly sharing data or code —e.g. participant privacy or use of data from a third party—those must be specified.

Reviewer #1: Yes

Reviewer #2: Yes

Reviewer #3: Yes

PLOS authors have the option to publish the peer review history of their article (what does this mean?). If published, this will include your full peer review and any attached files.

If you choose "no", your identity will remain anonymous but your review may still be made public.

Do you want your identity to be public for this peer review? *For information about this choice, including consent withdrawal, please see our Privacy Policy.*

Reviewer #1: No

Reviewer #2: No

Reviewer #3: No

Figure Files:

While revising your submission, please upload your figure files to the Preflight Analysis and Conversion Engine (PACE) digital diagnostic tool, https://pacev2.apexcovantage.com. PACE helps ensure that figures meet PLOS requirements. To use PACE, you must first register as a user. Then, login and navigate to the UPLOAD tab, where you will find detailed instructions on how to use the tool. If you encounter any issues or have any questions when using PACE, please email us at figures@plos.org.

Data Requirements:

Please note that, as a condition of publication, PLOS' data policy requires that you make available all data used to draw the conclusions outlined in your manuscript. Data must be deposited in an appropriate repository, included within the body of the manuscript, or uploaded as supporting information. This includes all numerical values that were used to generate graphs, histograms etc.. For an example in PLOS Biology see here: http://www.plosbiology.org/article/info%3Adoi%2F10.1371%2Fjournal.pbio.1001908#s5.

Reproducibility:

To enhance the reproducibility of your results, we recommend that you deposit your laboratory protocols in protocols.io, where a protocol can be assigned its own identifier (DOI) such that it can be cited independently in the future. Additionally, PLOS ONE offers an option to publish peer-reviewed clinical study protocols. Read more information on sharing protocols at https://plos.org/protocols?utm_medium=editorial-email&utm_source=authorletters&utm_campaign=protocols
