# Supplementary material for: Generative AI extracts ecological meaning from the complex three dimensional shapes of bird bills
Source: PLoS Comput Biol. 2025 Mar 17;21(3):e1012887. doi: 10.1371/journal.pcbi.1012887 (PMC11940575; doi:10.1371/journal.pcbi.1012887)
Supplement: Attachment — Submitted filename: Dinnage_and_Kleineberg_R2_Response_to_Reviewers.pdf [file pcbi.1012887.s006.pdf]

Response to Editor and Reviewers

Dear Dr. D'Andrea,

Thank you for the thoughtful reviews of our manuscript "Generative AI helps extract ecological meaning from the complex three dimensional shapes of bird bills". We appreciate the constructive feedback and have made substantial revisions to address the concerns raised. The major changes include:

1. Comprehensive analysis of phylogenetic signal in the latent space using PACA (Phylogenetically Aligned Components Analysis), demonstrating that DeepSDF naturally distributes phylogenetic signal uniformly across dimensions
2. Updated classification methodology using case weights to properly handle class imbalance
3. Improved PCA comparison using matched dimensionality (64 components) and preprocessing
4. Revised language regarding foundation models to better reflect the current state and limitations of the approach
5. Added detailed discussion of latent space properties, addressing non-deterministic nature and robustness
6. Completed the truncated GLM paragraph with full results

Below we address each comment from the editor and reviewers in detail.

Editor Comments

My apologies for the slow review process. It has proven difficult to find reviewers for this paper after two of the original reviewers became unavailable. One reviewer of the original manuscript, one new reviewer, and I have now assessed the revised manuscript. I appreciate the effort put into this revision, which in my view has substantially improved the manuscript.

Thank you for managing the review process. We appreciate the care taken to find appropriate reviewers for this interdisciplinary work.

Specifically, concerns were raised about the robustness and interpretability of the latent space, the potential phylogenetic structure of the data, and the appropriateness of labeling DeepSDF as a foundational model.

We have thoroughly addressed these concerns through:

1. A new section discussing latent space properties that compares DeepSDF's non-deterministic nature to other widely-used methods in biological data analysis such as NMDS
2. Comprehensive analysis of phylogenetic structure using PACA methodology
3. More measured language regarding the potential for foundation models in morphological analysis

Reviewer Comments

Reviewer #1

I thank the authors for engaging with my initial set of comments on their manuscript. Overall I feel that the authors have addressed my points satisfactorily, and I particularly welcome the additional analysis investigating the lower beak issue and the comparison of results to PC scores. Overall I think the revised manuscript is much improved compared to the original and I have no further points to raise.

We thank the reviewer for their positive assessment.

Reviewer #4

One of my primary concerns with this paper is the claim that non-linear dimension reduction represents a significant step forward for complex shape evolution when essentially none of the results support that claim. Unless I misunderstood, it seemed that PCA generally preformed better than or equal to DeepSDF across most tests.

Furthermore, the authors do not mention several ways in which PCA may be preferable to non-linear techniques. I believe the manuscript generally treats the problem in a one-sided way in which the non-linear techniques are always going to be better than linear counterparts. However, this is based on intuition rather than any theoretical prediction.

We thank the reviewer for this important point. We have revised the manuscript to present a more balanced view of the trade-offs between linear and non-linear approaches. Our new results show that DeepSDF and PCA achieve similar performance in many tasks, but with DeepSDF generally performing slightly better. We now emphasize that DeepSDF offers complementary benefits rather than strict improvements, particularly:

- The ability to work directly with raw 3D meshes, reducing the need for manual landmark placement
- Built-in regularization that helps handle noisy or imperfect data
- Natural distribution of phylogenetic signal, as demonstrated by our new PACA analysis
- The potential for transfer learning and model reuse

We updated our method for PCA in an attempt to make the comparison more fair, and because we needed to calculate PCA ourselves rather than using the 8 PC axes from Cooney et al. (2017), so that we could perform phylogenetic PCA. Firstly, we used the same alignment method on the original landmark data from Cooney et al as we used for aligning our meshes. Secondly, we used the first 64 PC axes to match the number of axes used in our DeepSDF analysis. It is likely one of these changes that led to the new result that PCA performs (very slightly) worse than DeepSDF (either the very basic alignment method resulted in poorer variables, or the greater number of variables increased overfitting). In addition we also updated our Random Forest Classification to use case weights in order to deal with unbalanced data, instead of the data augmentation approach we used previously based on upsampling low sample classes through the VAE generative model. We decided to do this to make sure we were not introducing any additional classification power for through this second model, for either approach. The reasoning being we wanted the original variables to 'stand on their own'. This change may have also led to our new result, where DeepSDF narrowly beats PCA (and overall the classification accuracy was lower). Nevertheless, the difference between the methods was still very small, so we have tried to keep our discussion of this result careful and measured.

1) The authors can correct me if I'm wrong, but generally these deep neural networks are not deterministic in the way that they model the latent space. This means that each time the model is run, there is potential for the latent space to be different. Besides the issues of reproducibility, this is potentially problematic if the latent spaces differ significantly each time the model is trained. How robust is this latent space simply to stochasticity in training? Are distances between points maintained when the model is trained?

We thank the reviewer for raising this important point about the non-deterministic nature of the latent space. We have added a new section discussing this property, which is actually shared with other widely-used methods in biological data analysis, such as NMDS, t-SNE, and UMAP. While methods like PCA produce deterministic results, they achieve this by making strong assumptions about linearity and orthogonality that may not reflect biological reality. The non-deterministic nature of more flexible methods represents a reasonable trade-off for their ability to capture complex non-linear relationships.

Previous work has extensively validated the robustness of DeepSDF and similar approaches (Park et al. 2019, citations). While different training runs may produce different exact coordinates, what matters is that the method reliably captures meaningful biological variation and relationships between specimens. Rather than conducting multiple computationally expensive training runs to demonstrate this directly, we focus on showing that our single trained model captures biologically meaningful variation through multiple lines of evidence: interpretable shape vectors, robust prediction of trophic niches, and biologically meaningful arrangement of species in latent space.

2) What exactly is being modeled in the latent space. One of the conveniences of PCA is that the principal components are generally easily defined by the input features. This is more complex for shape data, but it should be true that we can get insight into the latent PC space numerically and have an idea of the amount of variation explained when reducing the dimensionality. Is this possible for the latent space produced by an autoencoder?

The reviewer raises an excellent point about latent space interpretability. While DeepSDF's latent dimensions are not directly interpretable like PCA components, we can still gain insight into the space through multiple approaches:

1. Direct visualization of shape reconstructions demonstrates the quality of dimension reduction (Figure 1)
2. We can find interpretable directions through regression against known shape features (e.g., our elongation and broadness vectors)
3. Our new phylogenetic analysis shows the latent space effectively encodes evolutionary relationships, with remarkably uniform distribution of signal across dimensions
4. The success of the decoder in reconstructing shapes indicates meaningful compression rather than memorization

Rather than explaining variance along fixed orthogonal axes like PCA, DeepSDF learns a distributed representation where meaningful shape variations often involve combinations of latent dimensions. This can actually be advantageous - when we explicitly search for interpretable directions (like elongation), they can in some cases be more reliably extracted than from PCA components because they're not constrained to be orthogonal.

3) PCA has methods for phylogenetic correction which allow for the partitioning of variation due to common ancestry and variation in morphology. I realize the authors discuss future plans to extend this work with a phylogenetic component, but there is no mention of this assumption prior to that point in the discussion. How do we know that the latent space presented is not simply a reflection of the phylogeny?

This important concern prompted our new comprehensive phylogenetic analysis. Using PACA (Collyer & Adams 2021), we found that:

1. DeepSDF and PCA show similar overall levels of phylogenetic signal (multivariate $K = 0.16$ and 0.23 respectively)
2. DeepSDF naturally distributes this signal uniformly across dimensions, while PCA concentrates it in early axes

3. Classification performance remains strong even when using phylogenetically aligned components with the most phylogenetically informative axis removed, demonstrating that the latent space captures more than just phylogenetic structure
4. The ability to maintain predictive power while handling phylogenetic signal suggests DeepSDF learns a representation that naturally separates phylogenetic structure from other sources of variation

Another claim which I think needs to be addressed is the classification of this as a foundation model. The concept of foundation models lacks a clear definition, probably because it was essentially a corporate marketing strategy. Nonetheless, so-called "foundation" models do serve as a basis for many fine-tuned applications and are incredibly useful for AI applications. In contrast to the training in this paper, foundation models are trained on extensive datasets (millions of samples if not more), often carefully curated to ensure wide-ranging applicability. In my opinion, 2000 samples is not enough for this model to be labeled as foundational.

We appreciate this important critique and have significantly revised our claims regarding foundation models. The reviewer is correct that our current implementation, trained on ~2000 bird beaks, does not meet the scale typically associated with foundation models. We now present this work as an early exploration of how shared, reusable AI models might eventually be developed for morphological analysis. We acknowledge that true foundation models require massive, diverse datasets and extensive validation across multiple tasks. While we believe this approach shows promise, significant work remains to develop appropriate architectures, training approaches, and validation methods for true morphological foundation models.

Line 313: "potential is enormous". Based on what evidence do you make this claim. Deep learning is of course an incredibly powerful tool. But the model presented here was only able to match PCA. Would you say that PCA has enormous potential?

We have moderated this language. The revised text now reads: "The method shown here represents a promising new approach for analyzing 3D morphological data, though significant development work remains." We focus on specific demonstrated advantages while acknowledging current limitations and the need for further development.
