# Supplementary material for: Generative AI extracts ecological meaning from the complex three dimensional shapes of bird bills
Source: PLoS Comput Biol. 2025 Mar 17;21(3):e1012887. doi: 10.1371/journal.pcbi.1012887 (PMC11940575; doi:10.1371/journal.pcbi.1012887)
Supplement: Attachment — Submitted filename: Dinnage_and_Kleineberg_2025_R3_Response_to_Reviewers.pdf [file pcbi.1012887.s007.pdf]

Response to Editor and Reviewers

Dear Dr. D'Andrea,

Thank you for the opportunity to revise our manuscript "Generative AI helps extract ecological meaning from the complex three dimensional shapes of bird bills", in response to Reviewer #4's final concern. We have responded to their comments (in grey italics) below:

Comments to the Authors:

Reviewer #4: I thank the authors for their thorough response to my previous comments. I believe the more detailed PCA analysis and examination of phylogenetic signal represent a step forward in our understanding of non-linear latent spaces in the context of phylogenetic relationships.

Thank you for taking the time to review our revisions, and we are pleased that you found them satisfactory

My only concern remains classifying this as the first morphological "foundation model." The amount of data used to train a foundation model is many orders of magnitude greater than what was used in this study. For example, the recent sequence foundation model (Nguyen et al. 2024) and ct foundation model (Google research 2024) were trained on 2.7 million sequences and 3.7 million image scans respectively. These are foundation models because their extensive training allows the model to potentially gain some higher level understanding of their task. This higher level understanding then allows the foundation models to preform zero-shot learning and be used as a base for future fine tuning. 2,000 data samples is simply not enough for this model to be classified as a foundation model. The authors should remove claims that this is a foundation model. It is not necessary to include in this manuscript and, in my opinion, it is a false claim.

We very much agree with this perspective. After considering your previous review we decided to remove the claim that our model was a foundation model, and instead discuss it as an early step along the path to a possible foundation model in the future in the main manuscript (e.g. exploring possible architectures for morphological AI models). Unfortunately, it appears that we neglected to remove the foundation model claim from the Abstract in the submission system. This was an unfortunate oversight, which was not intended. We have now made sure the Abstract has also been updated to make no claim of foundation model status. There is now no claim of foundation model status anywhere in the manuscript, though we still retain a discussion of foundation models as a long-term goal of this research direction, since we believe that readers will be interested in this idea.

Nonetheless, this is an exciting proof of concept! With much additional training data, this model could prove to be a foundation model (though, in truth, I suspect a more complex architecture will be necessary to take advantage of larger datasets). And other than the claim of DeepSDF as a foundation model, I have no issues with the manuscript as is. I am excited to see this work progress and the advances that can be made in this area.

Thank you for saying so. We agree that this should serve as a proof of concept and hope that we, along with others, can quickly build on this progress to create something more truly foundational in the near future.
