# Supplementary material for: Generative AI extracts ecological meaning from the complex three dimensional shapes of bird bills
Source: PLoS Comput Biol. 2025 Mar 17;21(3):e1012887. doi: 10.1371/journal.pcbi.1012887 (PMC11940575; doi:10.1371/journal.pcbi.1012887)
Supplement: Attachment — Submitted filename: Review.docx [file pcbi.1012887.s003.docx]

**Comments to the Corresponding Author**

This manuscript employs a deep generative modelling approach called DeepSDF to visualize and analyze beak shape variation across different species. The authors additionally show how the latent morphology data can be used in various downstream analyses, such as classification. The main contribution of this work is the application of a slightly modified DeepSDF method to generate morphological data. The authors show via dimensionality reduction methods that the latent data result in clustering of trophic niches. They also report that their random forest classification model was able to correctly predict the trophic niche of most birds. The figures summarized these tests well and were expertly put together.

While the modeling framework and visualizations were interesting, I have several major concerns. I was not actually sure what the novelty or overall objective of this paper was. On the one hand, the manuscript comes across as attempting to introduce a refined DeepSDF method, although most of the math and formulae shared throughout the work are taken from the original paper. If there was a methodological angle, a lot more needed to be presented. For example, how does this method perform on different sample sizes, or maybe different organisms, as opposed to a (relatively large) sample of 2000 bird beak meshes? And where are the loss curves or discussions of reconstruction error? On the other hand, it is trying to argue that the learned latent data have increased ecological meaning, but this is not explicitly tested relative to another method. As someone unfamiliar with bird anatomy and their classification accuracies, it was not clear to me whether this approach actually outperforms others. In other words, the findings do not actually support the claims.

The other issue I had was the overall presentation of the manuscript. There were a number of spelling and punctuation issues, the writing was not concise, and it was not organized according to the journal’s policies. These problems can obviously be fixed, but as is, they greatly hindered the reading.

**Minor comments**

Lines 44-60: Talking about development provides a set of instructions to assemble a phenotype through non-linear probabilistic dynamics; development as a non-linear decoder. Strong argument.

Line 61: A.I. acronym should be artificial intelligence (AI).

Line 73: three-dimensional (3D).

Line 75: I agree with these challenges.

Line 80: 3D not 3d.

Line 100-102: demonstrate the utility of deep generative models? What does that mean?

Lines 103-112: This paragraph comes out of nowhere and should be better integrated into the introduction. Details of the dataset should be provided below in the Materials and Methods section.

Line 113: Results should follow the Introduction, as per the journal guidelines, correct?

Lines 115-117: Even though the dataset is publicly available, there should at least be some details on the specific scanner, how it acquires images, the surface reconstruction, etc.

Line 119: The authors don’t clarify what packages, etc. were used in the “algorithm written in R” until line 142. Either state your completed the following steps: “…” etc., or clarify what packages were used upfront.

Lines 123-131: This reorientation step, where the authors use existing landmarks to align the data, is not as trivial as the authors suggest. Landmark-free (i.e., data-driven surface or volumetric registration) methods are often error-prone and difficult to implement.

Line 128: Extra spacing? And this website is repeated too much.

Line 145: Again, 3D, not 3 dimensional.

Line 152-153: Confusing wording.

Lines 167-187 (Model Specifics): The math is important but it seems overkill here, especially on pg. 8, as the authors are not introducing a new method. They are simply restating formulae from the original Park et al. (2019) signed distance function paper.

Line 177: 3D instead of 3d. Nitpicking, but this should be consistent throughout the paper.

Line 181: Why was δ set to 0.1? Was this tested empirically?

Line 189: Wording? “Note that using”?

Line 191: Contraction.

Line 196: Spacing.

Line 200: I would argue a dataset of 2,000 bird beaks is very large for a biological study, not relatively small.

Line 202-203: Introduce VAE acronym?

Line 215: Spacing.

Lines 205-213: They use a modified neural net that appears to improve performance in Park et al. (2019).

Line 215: Spacing.

Lines 214-225: Training the DeepSDF model. Do they have any figures of the loss?

Lines 234-243: DeepSDF implemented in PyTorch for Python; model has another R implementation using the torch package? Bouncing between R and Python.

Line 256: “in our case”

Line 259: Spacing.

Lines 259 to 261: Talking about two-stage VAEs and fitting a second-stage VAE to the first stage DeepSDF latent vector data, then they say the second-stage VAE estimated latent space conformed strongly to an independent Gaussian distribution, whereas the first latent vectors deviated substantially? Where are the results/figures?

Lines 270-287: They start discussing VAEs at length here. Why not do this at the beginning of the section?

Lines 297-298: Wording? “Using common machine learning algorithms”?

Line 346: Wording? “Were”?

Line 348: Wording? “Well-known”?

Lines 353-356: Why not just upsample or downsample? Why use a combination? Were you trying to land on a specific number of observations per class?

Line 358: Punctuation.

Line 365: Wording? “Revealed”?
Line 367: What downstream tasks?

Line 368: Is there any discussion of reconstruction error anywhere? I don’t see it in Fig. 1.

Lines 373 to 382: I don’t understand why there needs to be a discussion about ‘entanglement’. The authors are basically just hinting at morphological integration, or the tendency for structures to covary. This is a ubiquitous feature of organisms due to shared developmental pathways, so one would actually expect (and hope to see) how features are ‘entangled’.

Lines 386-388: I would like to see how UMAP compares to standard linear dimensionality reduction methods (e.g., PCA/CVA and PLS) with these data.

Lines 392-406: It’s interesting to see these classification results, but there’s no comparison. For example, what classification accuracies would you get with linear measurements, landmarks, etc.?

Line 405: Just say most broad or fat-tailed instead of most ‘diffuse’? Odd phrasing.

Lines 427-447: This is far too lengthy. Classification is not the main point of this paper. Suggesting one or two likely reasons for omnivore misclassification seems sufficient.
